# An Experimental Sequential Digestion Method for Efficient Isolation of Human Adipose-Derived Microvascular Fragments with Enhanced Angiogenic Potential

**DOI:** 10.1007/s00266-025-05606-0

**Published:** 2026-01-21

**Authors:** Xiya Yin, Xiangqi Liu, Jing Yang, Xingran Liu, Qiumei Ji, Yun Xie, Gang Chen, Qingfeng Li, Ru-Lin Huang

**Affiliations:** 1 Department of Plastic and Reconstructive Surgery, Shanghai Ninth People’s Hospital, Shanghai Jiao Tong University School of Medicine, 639 Zhizaoju Road, Shanghai, 200011 China; 2 Shanghai Institute for Plastic and Reconstructive Surgery, Shanghai Ninth People’s Hospital, Shanghai Jiao Tong University School of Medicine, Shanghai, 200011 China

**Keywords:** Microvascular fragments, Human adipose tissue, Vascularization, Enzymatic digestion, Tissue engineering, Regenerative medicine

## Abstract

**Background:**

Microvascular fragments (MVFs) are intact vascular segments derived from adipose tissue that possess considerable potential for promoting tissue vascularization in regenerative medicine. However, conventional single-step enzymatic digestion methods often lead to incomplete adipose tissue dissociation and poor MVF quality.

**Methods:**

We developed and validated a sequential enzymatic digestion protocol optimized for isolating MVFs from human lipoaspirate. Adipose samples were processed using either a conventional one-step collagenase digestion or a three-step sequential method. MVFs were evaluated for yield, viability, structural integrity, cellular phenotype, and angiogenic function both in vitro and in vivo.

**Results:**

Compared with the conventional approach, the sequential protocol produced a 2.2-fold increase in MVF yield and significantly reduced undigested tissue residues (p < 0.0001). MVFs isolated by the sequential protocol showed superior cell viability (93.3% vs. 75.6%), a greater proportion of long fragments, preserved endothelial and perivascular architecture, and enhanced angiogenic performance in collagen gel assays and mouse subcutaneous implantation models.

**Conclusions:**

This optimized sequential digestion protocol enables the efficient and producible isolation of high-quality MVFs from human adipose tissue. It holds great promise for applications in vascularized tissue engineering and regenerative therapies.

**No Level Assigned:**

This journal requires that authors assign a level of evidence to each submission to which Evidence-Based Medicine rankings are applicable. This excludes Review Articles, Book Reviews, and manuscripts that concern Basic Science, Animal Studies, Cadaver Studies, and Experimental Studies. For a full description of these Evidence-Based Medicine ratings, please refer to the Table of Contents or the online Instructions to Authors www.springer.com/00266.

**Graphical Abstract:**

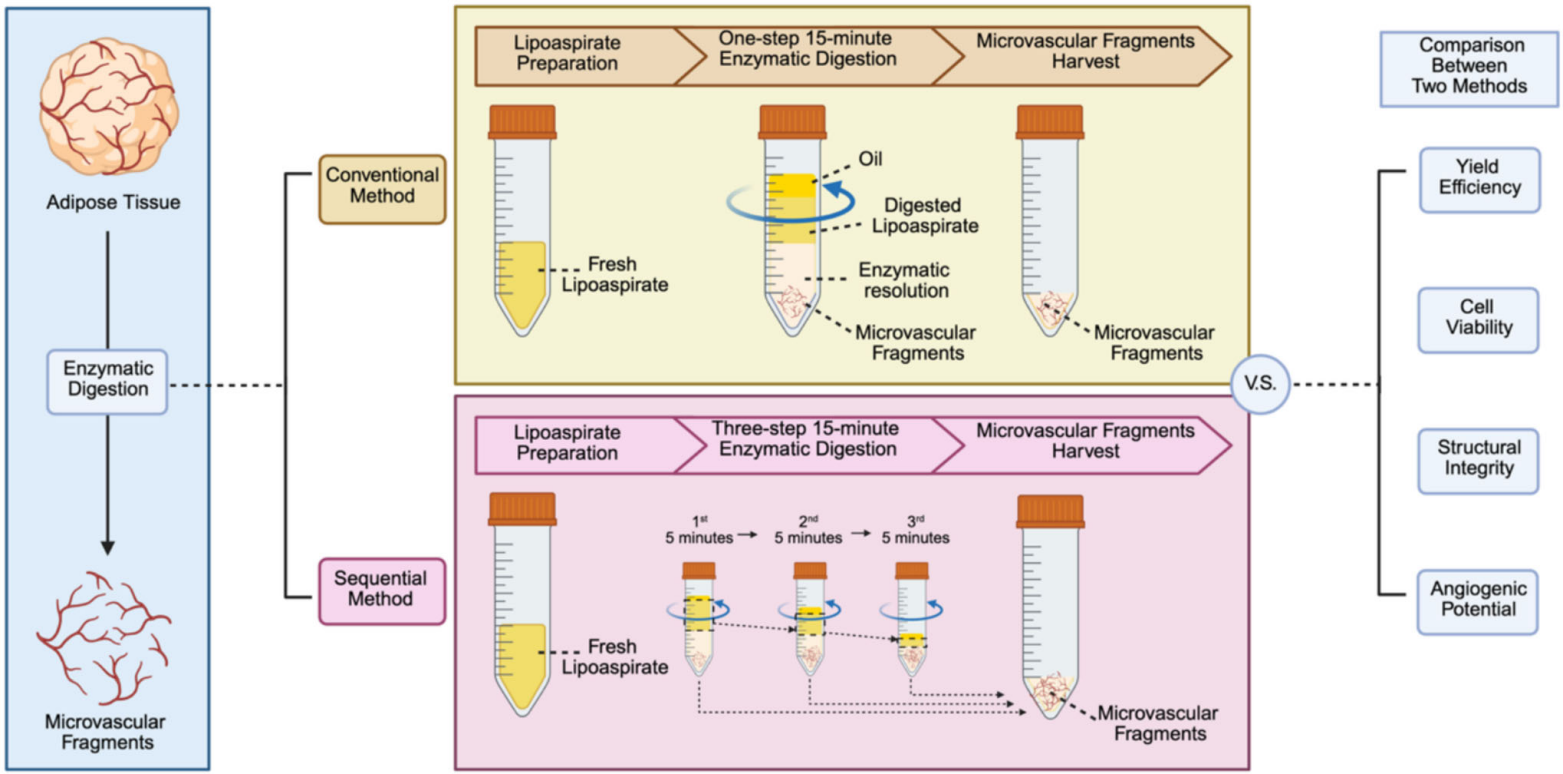

## Introduction

Vascularization is essential for tissue survival, regeneration, and function, particularly under ischemic conditions where inadequate blood supply leads to necrosis and impaired healing [1, 2]. In tissue engineering, promoting rapid and functional vascularization remains a major challenge, as many current strategies result in delayed perfusion, leaky vessels, and poor graft integration [3–5].

Microvascular fragments (MVFs), derived from adipose tissue through enzymatic digestion, have emerged as a promising vascularization units. Unlike stromal vascular fraction (SVF), MVFs consist of intact microvessel segments, including arterioles, venules, and capillaries, preserving the native multicellular architecture [6–8]. As they retain the cellular complexity and structural integrity of blood vessels, MVFs can rapidly inosculate with host vasculature within days upon implantation, promoting early perfusion and graft survival [9, 10]. In addition, MVFs harbor perivascular cells and mesenchymal stem cells (MSCs), contributing to tissue regeneration and angiogenesis via paracrine singles [11]. These features make MVFs highly relevant for applications requiring rapid vascular integration, such as accelerating wound healing [12, 13], treating ischemic disease models [9, 14, 15], generating prevascularized tissue constructs [16–22], and promoting tissue graft survival [23, 24], demonstrating their clinical potential.

The standard approach for isolating MVFs involves a single-step enzymatic digestion using collagenase [25, 26]. However, this method presents several limitations when applied to human adipose tissue. Continuous exposure to collagenase may result in either incomplete digestion, leaving large amount of residual tissue, or over-digestion, which breaks MVFs down into single cells like SVFs and reduce viability due to collagenase-induced cytotoxicity and microvascular structures. These limitations are particular prominent when using heterogeneous clinical lipoaspirates, which vary in fat content and fibrous composition compared to rodent epididymal fat pads traditionally used for protocol development [25, 27, 28].

To address these limitations, we developed a sequential enzymatic digestion protocol specially tailored for human lipoaspirate. By dividing the digestion processes into several short cycles, this method aims to minimize over-digestion and optimize MVF recovery. Here, we systematically compare the sequential and conventional methods in terms of MVF yield, structural integrity, viability, phenotype, and angiogenic potential both in vitro and in vivo. Our study provides a practical and efficient protocol for isolating MVFs from human adipose tissue, with potential applications in prevascularized tissue constructs and clinical regenerative therapies. This method may serve as a foundation for standardized, clinically compatible protocols for vascularized tissue engineering applications.

## Materials and Methods

### Human Adipose Tissue Samples

Between March and June 2024, lipoaspirate samples were collected from 12 healthy adult female donors undergoing liposuction procedures at Shanghai Ninth People’s Hospital, Shanghai Jiao Tong University School of Medicine. The donors had a mean age of 32.9 ± 4.5 years (range: 24–40 years) and a mean body mass index (BMI) of 25.48 ± 3.27 kg/m^2^ (range: 21.22–32.02 kg/m^2^), representing a cohort with generally normal-to-mildly overweight body habitus. Donor characteristics are summarized in Table 1.

**Table 1:** Human adult adipose tissue donors used in this study

Donor ID	Gender	Age (years)	BMI (kg/m^2^)	Location
A122	Female	37	23.05	Thigh
A123	Female	37	32.02	Abdomen
A124	Female	25	23.71	Abdomen
A125	Female	39	25.23	Abdomen
A127	Female	22	21.22	Abdomen
A130	Female	38	29.41	Abdomen
A137	Female	36	23.81	Abdomen
A142	Female	35	30.47	Abdomen
A143	Female	42	21.97	Abdomen
A145	Female	22	23.88	Abdomen
A151	Female	24	22.89	Abdomen and thigh
A154	Female	38	24.47	Abdomen

All procedures were conducted with written informed consent from each participant. The collection, processing, and experimental use of human adult adipose tissue samples were performed in full accordance with national regulations and institutional guidelines for human biomedical research in China. All protocols involving human tissue were reviewed and approved by the Institutional Review Board of Shanghai Ninth People’s Hospital, Shanghai Jiao Tong University School of Medicine (SH9H-2025-T306-1) and was carried out in accordance with the Declaration of Helsinki. Data were processed in compliance with applicable data privacy regulations.

### Animals

A total of 9 BALB/c nude mice (age: ~6 weeks, weight: 18–24 g) were purchased from Shanghai Jihui Experimental Animal Breeding Farm. All animal procedures were approved by the Institutional Animal Care and Use Committee of Shanghai Ninth People’s Hospital (approval number: SH9H-2023-A784-1). Mice were maintained under standard housing conditions (20–26 °C, 40–70% relative humidity, 12-h light/dark cycle) with ad libitum access to food and water.

### MVF Isolation Procedure

**Fig. 1:**
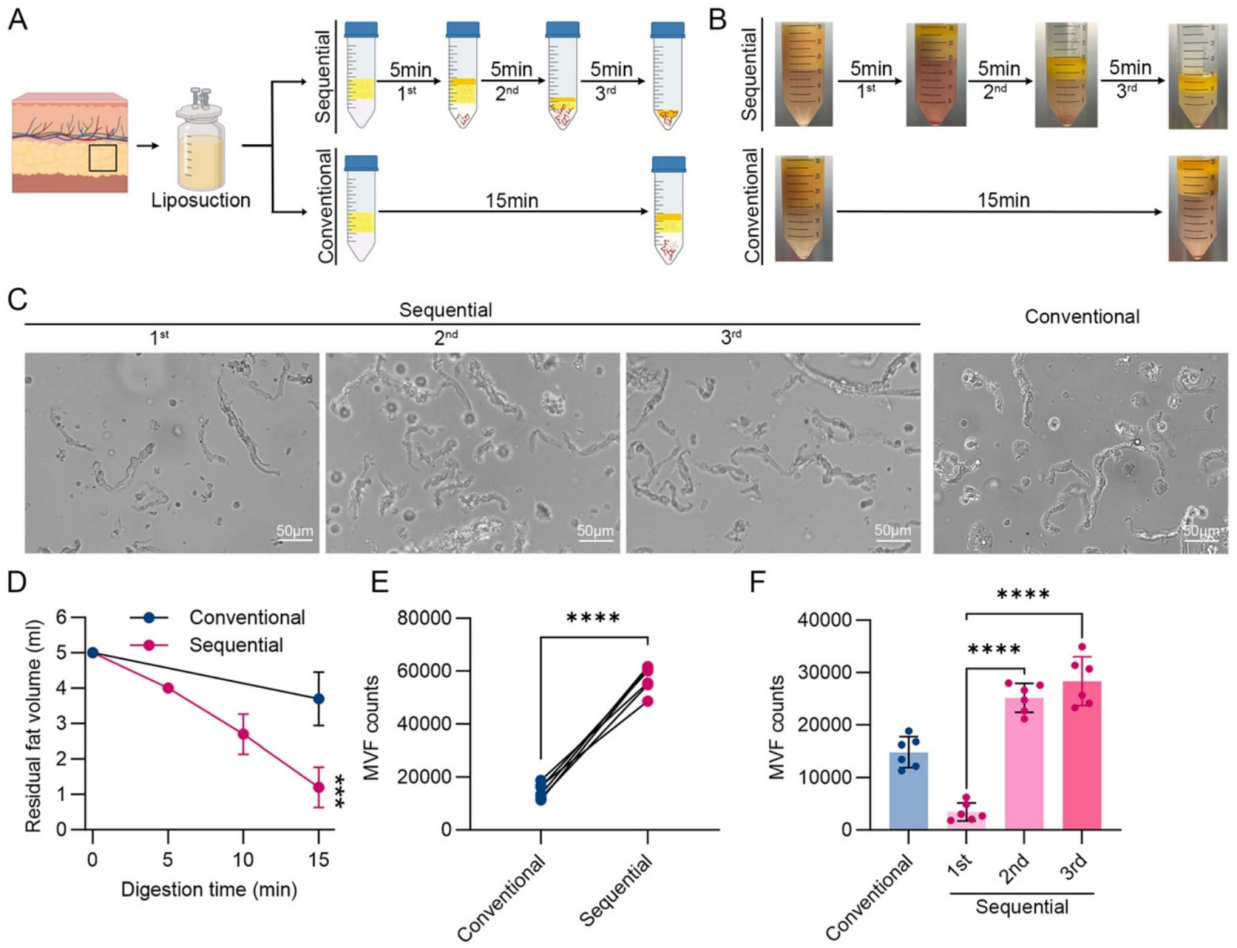
The sequential digestion method improves MVF yield efficiency compared to the conventional single-step method. **A** Schematic diagram comparing the conventional and sequential digestion protocols for isolating MVFs from human lipoaspirate. **B** Representative images of residual undigested adipose tissue after each digestion stage (5, 10, and 15 min) in both methods. **C** Representative phase-contrast images of MVFs collected after each digestion cycle. **D** Residual volume of undigested adipose tissue after each digestion stage (5, 10, and 15 min) in both methods. **E** Total MVF yield per milliliter of adipose tissue after complete digestion using each method (*n* = 6), **** *p* < 0.0001. **F** MVF yield per cycle in the sequential digestion method versus single-step digestion in the conventional method (*n* = 6), **** *p* < 0.0001

Lipoaspirate samples were washed with sterile phosphate-buffered saline (PBS), mechanically dissected using fine scissors, and divided into two groups for MVF isolation using either the conventional or the sequential digestion method (Fig. 1A).

#### Conventional Method

Five milliliters of adipose tissue were mixed with 5 mL of 0.2% collagenase NB4G (Nordmark, German) in PBS at 37 °C for 15 min with continuous agitation. The enzymatic reaction was terminated by adding two volumes of 20% fetal calf serum (FCS; Thermo Fisher Scientific, USA) in PBS. The digested mixture was filtered through a 500-μm nylon mesh to remove residual adipose tissue, followed by a 30-μm mesh to collect MVFs. MVFs retained on the mesh were rinsed thoroughly with 20% FCS-PBS and centrifuged at 200 × g for 5 min. The pellet was resuspended in 1 mL of 20% FCS-PBS for further use.

#### Sequential Method

The same volume of adipose tissue (5 mL) was subjected to three consecutive digestion cycle. In each cycle, adipose tissue was incubated with 5 mL of 0.2% collagenase NB4G at 37 °C for 5 min with shaking. After each cycle, the suspension was filtered, washed, and centrifuged as described above to collect MVFs. Undigested residual tissue was used for the next digestion cycle. After the third cycle, only oil droplets remained, which were discarded.

### MVF Quantification and Length Analysis

MVFs were counted using a Neubauer chamber with a 1:40 dilution. Ten microliters of suspension were used for counting, and total yield was calculated accordingly. For length distribution analysis, 20 μL of MVF suspension was observed under a Leica microscope, and 100 MVFs per samples was measured using imaging software.

### Viability Assessment

Cell viability was assessed using the LIVE/DEAD^TM^ Viability/Cytotoxicity Kit (Thermo Fisher Scientific). MVFs were stained and analyzed by fluorescence microscopy. Viable (green) and dead (red) cells were counted, and viability was expressed as a percentage of total cells.

### Flow Cytometry

MVFs were enzymatically dissociated into single cells using Accutase (BioLegend, USA) for 30 minutes at 37 °C. Cells were stained with fluorescence-conjugated antibodies against human CD31 (Violet 605), CD146 (APC), CD45 (PerCP), CD90 (APC-A750), and CD73 (FITC) (all from BioLegend). Flow cytometry was performed with appropriate isotype controls. Data acquisition was performed on a BD FACScan cytometry (BD Biosciences, USA) and analyzed with FlowJo software (TreeStar, USA).

### In Vitro Angiogenesis Assay

MVFs from each method were suspended in neutralized type I collagen at a concentration of 20000 MVFs/mL. The final collagen solution (2 mg/mL) was dispensed into 48-well plates (200 μL per well) to allowed to gel. The MVF-containing collagen gels were cultured in DMEM with 10% FBS at 37 °C. Sprouting activity was monitored at days 3, 6, and 9, under brightfield microscopy. Sprouting area was quantified using ImageJ software.

### In Vivo Implantation of MVF-Seeded Constructs

Nine nude mice were randomly divided into three groups: a control group, a group receiving conventionally digested MVFs, and a group receiving sequentially digested MVFs. Three mice per group were used to balance preliminary study needs with ethical and practical constraints. MVFs were embedded in type I collagen gels (2 mg/mL, 20000 MVFs/mL) as above described. Control constructs without MVFs were prepared similarly. Each mouse was implanted subcutaneously with one construct on both sides of their flanks. A small incision was made, and constructs were inserted into subcutaneous pockets. The skin was closed using 6-0 sutures, and mice were allowed to recover.

### Histology and Immunohistochemistry

Constructs were harvested 14 days post-implantation, fixed in 4% paraformaldehyde for 12 hours, and processed for 5-µm paraffin sections. Hematoxylin and eosin (H&E) staining was performed using standard protocols.

For immunofluorescence, sections were permeabilized with 0.5% Triton X-100, blocked with 10% goat serum (Sigma-Aldrich, USA), and incubated overnight with primary antibodies: anti-human CD31 (1:200; Abcam, UK) and anti-human α-SMA (1:200; Abcam). After washing, slides were incubated with secondary antibodies (Alexa Fluor 488 and 546) and counterstained with DAPI (Sigma-Aldrich). Imaging was captured using a Leica Stellaris 8 Falcon microscope (Leica Microsystems, Germany), and analyzed using LAS X software (version 4.7, Leica Microsystems).

### Statistical Analysis

Data are presented as mean ± standard deviation (SD). Comparisons between two groups were made using unpaired Student’s t test. For comparisons among three or more groups, one-way ANOVA followed by Bonferroni post hoc tests was applied. Statistical significance was set at p < 0.05.

## Results

### Sequential Digestion Increases MVF Yield and Reduces Residual Tissue

To compare the efficiency of MVF isolation, 5 mL of human lipoaspirate was digested using either the conventional single-step method or the sequential three-step digestion protocol (Fig. 1A and B). Microscope confirmed the presence of vessel fragments in both groups (Fig. 1C). However, the volume of undigested residual tissue was significantly lower in the sequential group (1.2 ± 0.57 mL) than in the conventional group (3.7 ± 0.76 mL) (Fig. 1D).

Quantification revealed that the sequential method yielded (5.70 ± 0.50) × 10^4^ MVFs, which was approximately 2.2-fold higher than the conventional method’s yield of (1.48 ± 0.29) × 10^4^ MVFs (*p* < 0.0001) (Fig. 1E). Further breakdown of the three sequential digestions showed that the second and third cycles contributed to majority of the yield: (2.52 ± 0.10) × 10^4^, and (2.83 ± 0.16) × 10^4^ MVFs, respectively (Fig. 1F). These results suggest that the sequential digestion method more thoroughly dissociates adipose tissue and maximizes MVF recovery.

### MVFs Isolated by the Sequential Digestion Exhibit Greater Length and Viability

**Fig. 2:**
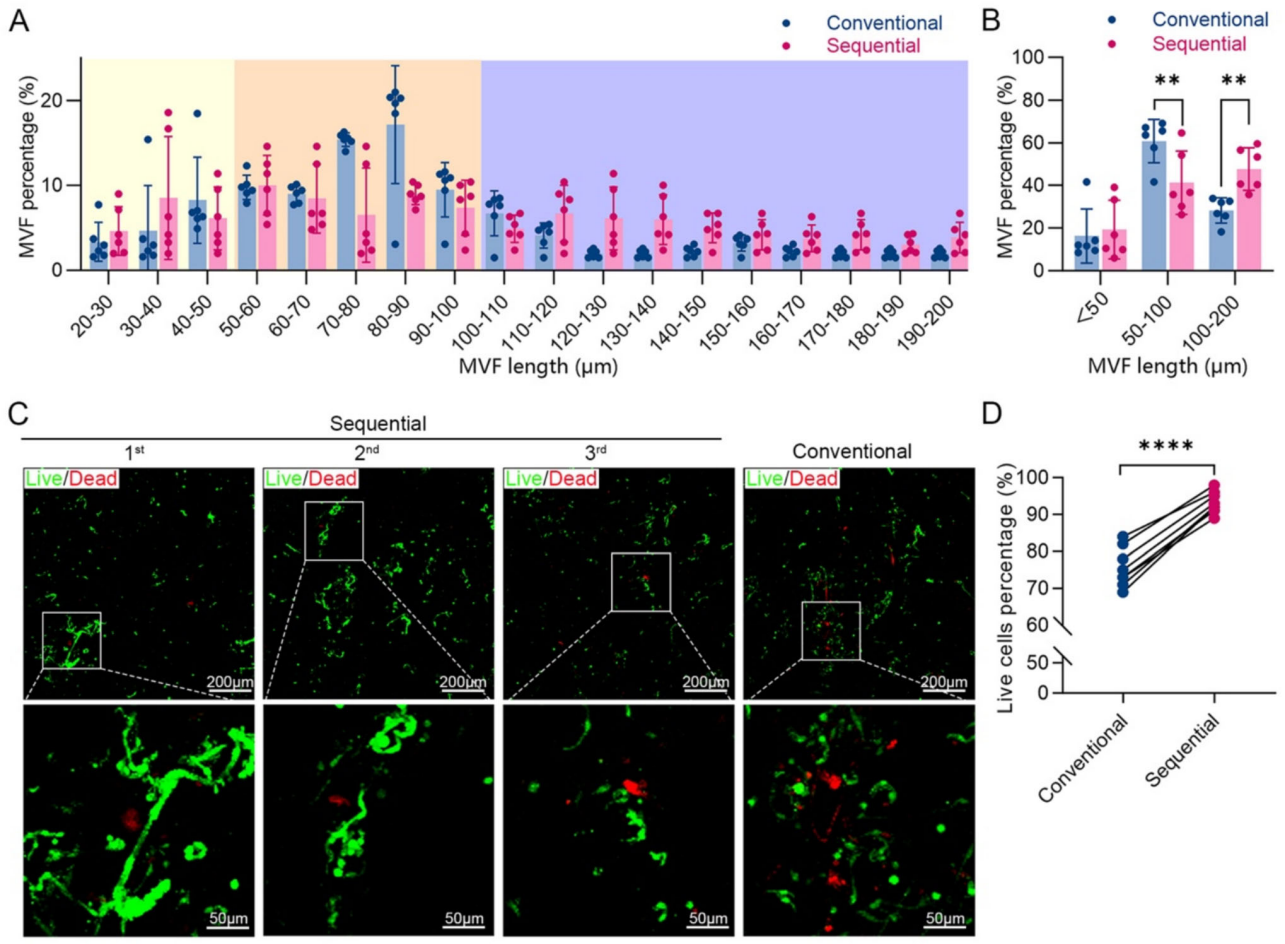
MVFs isolated via sequential digestion method exhibit longer fragments and improved cell viability. **A** Length distribution of MVF isolated by each method (*n* = 6). **B** Proportion of MVFs categorized as short (<50 μm), medium (50–100 μm), and long (100–200 μm) fragments (*n* = 6); ** p < 0.01. **C** Representative fluorescence images of live (green) and dead (red) cells within MVFs collected after each digestion cycle. **D** Quantification of cell viability (%) based on live/dead staining (*n* = 7); **** *p* < 0.0001

MVF length distribution differed significantly between the two methods. In the conventional group, 60.8 ± 10.2% of MVFs were 50–100 μm in length, while only 28.2 ± 5.9% were >100 μm. In contrast, the sequential group yielded 47.7 ± 10.0% long fragments and fewer middle-length fragments (41.3 ± 14.9%) (Fig. 2A and B). This shift toward longer MVFs suggests reduced over-digestion in the sequential method.

Viability was also markedly improved. Live/dead staining revealed fewer red-stained (dead) cells in the MVFs from the sequential method (Fig. 2C). Quantification showed a significantly higher viability in the sequential group (93.3 ± 1.0%) compared to the conventional group (75.6 ± 1.9%) (*p* < 0.0001) (Fig. 2D). These findings indicate that shorter, repeated digestion cycles preserve MVF structure and cellular integrity more effectively.

### Cellular Composition and Structural Integrity of MVFs Are Preserved

**Fig. 3:**
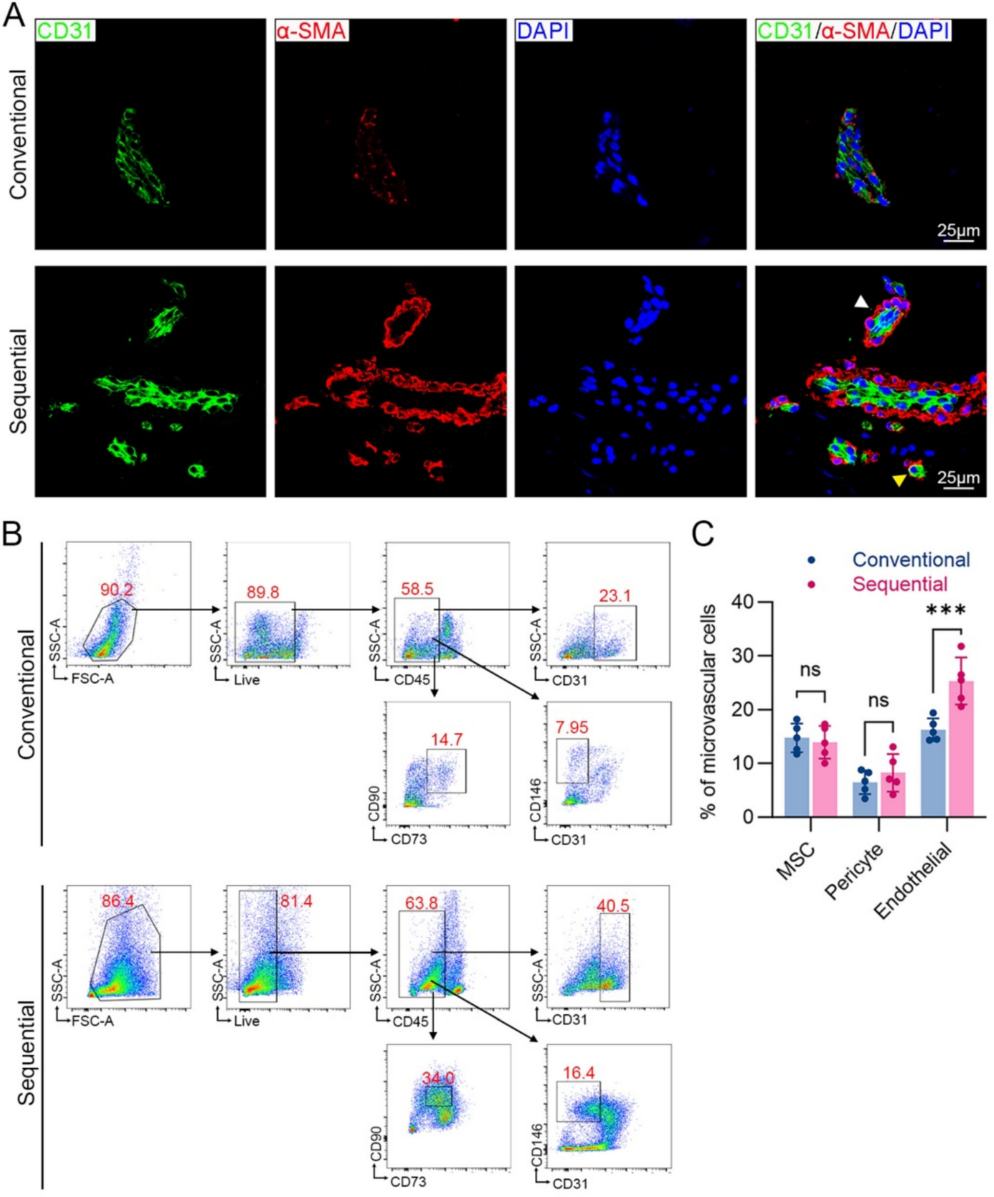
Cellular composition and structural integrity of MVFs are preserved with the sequential digestion method. **A** Representative immunofluorescence images of MVFs isolated using each method. Nuclei (blue), endothelial cells (CD31, green), and smooth muscle cells (α-SMA, red). Arterioles (white arrow) and capillaries (yellow arrow) are indicated. **B** Flow cytometry gating strategy for identifying pericytes, endothelial cells, and MSCs. **C** Quantification analysis of cell population in MVFs isolated by both methods (*n* = 5); *** *p* < 0.001, ns, no significant

It has been previously reported that MVFs contain MSCs, pericytes, endothelial, and smooth muscle cells [29]. To assess whether the sequential method affects MVF identity, we performed immunofluorescence and flow cytometry. Immunostaining showed that MVFs from both methods maintained typical vascular architecture, with intact CD31^+^ endothelial cells forming luminal rings and α-SMA^+^ smooth muscle cells surrounding the outer layer (Fig. 3A).

Flow cytometric analysis revealed comparable cell composition between the two methods. MVFs isolated via the sequential methods contained 13.1 ± 3.8% of MSCs (CD45^-^CD73^+^CD90^+^), 26.5 ± 4.9% endothelial cells (CD45^-^CD31^+^), and 7.63 ± 5.2% pericytes (CD45^-^CD31^-^CD146^+^), with no significant differences from the conventional method (Fig. 3B and 3 C). These results confirm that the sequential method does not alter MVF cellular phenotype or compromise vessel integrity.

### In Vitro Angiogenic Potential Is Enhanced in MVFs from Sequential Digestion

**Fig. 4:**
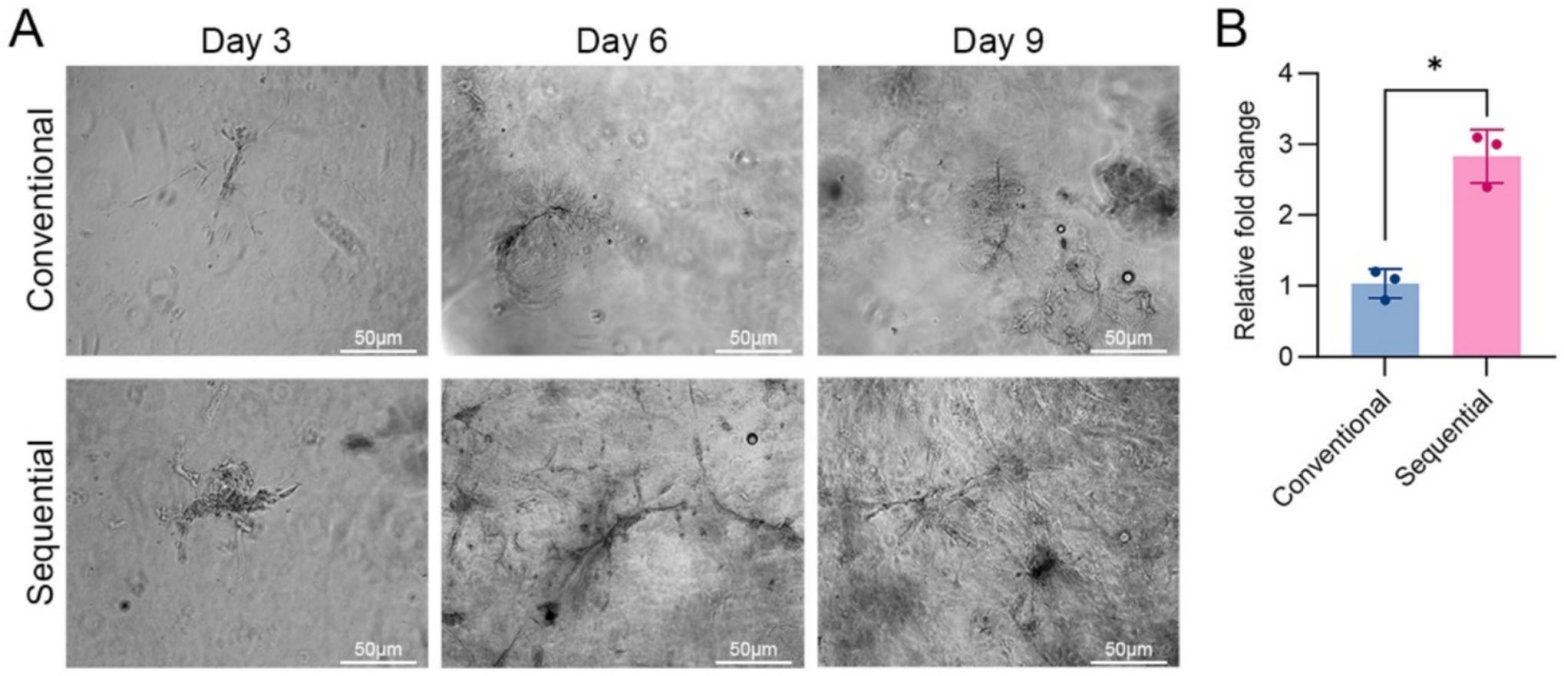
Sequentially isolated MVFs display enhanced angiogenic sprouting in vitro*.* **A** Representative phase-contrast images showing MVF sprouting in 3D collagen gels at days 3, 6, and 9 after culture. **B** Quantification of sprouting area at day 9 (*n* = 3); * *p* < 0.05

To evaluate functional activity, MVFs were embedded in type I collagen gels and cultured to assess sprouting behavior. Sprout formation began on day 3 in both groups. By day 6, MVFs from the sequential method showed noticeably more branching than sprouting structures (Fig. 4A). Quantification analysis on day 9 showed a significantly larger cumulative sprouting area in the sequential group (1.7-fold higher than that the conventional method; *p* < 0.05) (Fig. 4B), suggesting superior angiogenic capacity.

### MVFs from Sequential Digestion Promoted Vascularization In Vivo

**Fig. 5:**
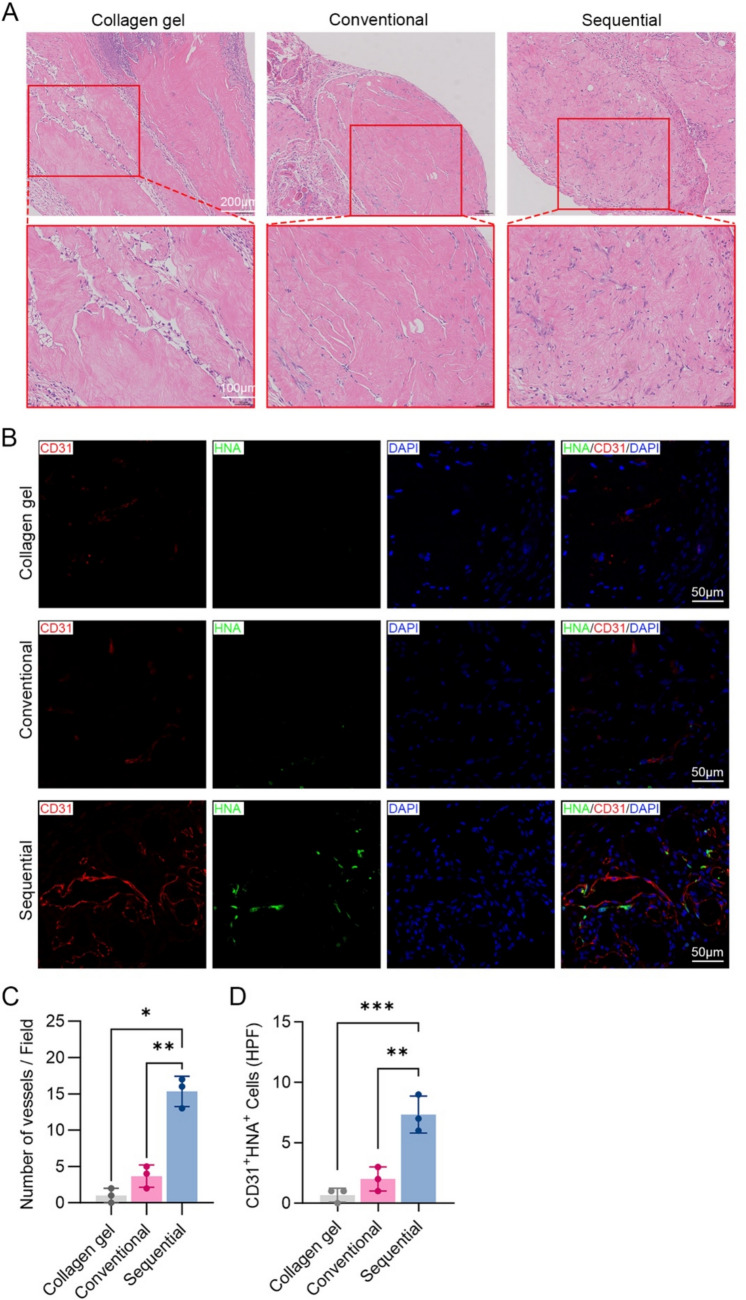
Sequentially isolated MVFs promote greater vascularization in vivo*.* **A** Representative H&E-stained sections of MVF-containing collagen gel implants harvested at day 14. **B** Quantification of blood vessel density based on H&E images (*n* = 3); * *p* < 0.05, ** *p* < 0.01. **C** Representative immunofluorescence images showing human-specific endothelial cells (CD31, red), human nuclei (HNA, green), and nuclei (DAPI, blue) in MVF-containing collagen gel implants harvested at day 14. **D** Quantification of perfused human-derived vessels (HNA^+^CD31^+^) per field (*n* = 3); ** *p* < 0.01, *** *p* < 0.001

To assess in vivo vasculogenic potential, MVF-containing collagen gels were subcutaneously implanted into nude mice. After 14 days, H&E staining revealed more vessel-like structures in both groups. Quantification confirmed a significantly higher microvessel density in this group compared to the conventional method (*p* < 0.01) (Fig. 5C). Immunofluorescence staining for human nuclear antigen (HNA) and CD31 further demonstrated increased formation of perfused, human-origin vessels in the sequential group (Fig. 5B and D). These findings support the enhanced vascularization potential of MVFs isolated by the optimized protocol.

## Discussion

In this study, we established a sequential enzymatic digestion protocol that significantly improves the isolation efficiency and quality of MVFs from human adipose tissue. Compared to the conventional single-step method, the sequential protocol yielded over two-fold more MVFs, exhibited higher cell viability, and preserved greater structural integrity. These MVFs demonstrated superior angiogenic potential both in vitro and in vivo, highlighting the translational promise of this refined method.

This work addresses a key challenge in current MVF-based tissue engineering strategies: the lack of a standardized and efficient protocol for isolating human MVFs. Most established protocols have been developed using murine epididymal fat pads [25, 27], subcutaneous fat [27, 30] or brain vasculature [31], which differs substantially from human subcutaneous adipose tissue in terms of extracellular matrix (ECM) density and fibrous content. As a result, conventional digestion protocols often result in either incomplete dissociation, leaving a large portion of the adipose tissue unprocessed, or over-digestion, which breaks down MVFs into SVF single cells and compromises structural and functional integrity.

Our sequential digestion protocol, based on three consecutive short enzymatic cycles, offers a flexible and controlled method to address these limitations. We demonstrated that this method not only reduced residual undigested tissue but also increased the total MVF yield by 2.2-fold while maintaining vascular segment length and cell viability. Importantly, this improvement was achieved without prolonging the overall digestion time, but rather by timely separating MVFs from the collagenase solution after each cycle, thereby avoiding excessive enzymatic degradation. Live/dead staining confirmed that cells within MVFs isolated by the sequential method were significantly more viable than those from the conventional group, and structural analysis showed a higher proportion of long fragments and preserved vessel-like morphology.

Another important advantage of the sequential approach is its scalability and adaptability. Because each digestion cycle is followed by intermediate assessment and separation, the number of digestion rounds can be dynamically adjusted based on the degree of tissue dissociation. This feature allows the protocol to be tailored to different fat qualities, such as more fibrous samples or smaller biopsy volumes, ensuring complete and efficient MVF recovery without compromising viability or structure. Such flexibility makes the method especially suited for handling heterogeneous clinical samples and facilitates translation into diverse experimental or therapeutic contexts.

Our results further showed that the sequential protocol maintains the cellular composition of MVFs, including endothelial cells (CD45^−^CD31^+^), pericytes (CD45^−^CD31^−^CD146^+^), and MSCs (CD45^−^CD73^+^CD90^+^), as validated by immunofluorescence and flow cytometry. These MVFs exhibited robust angiogenic sprouting in vitro and formed functional, perfused vessels in vivo, with significantly increased HNA^+^CD31^+^ microvessels in mouse subcutaneous implants.

The enhanced quality of MVFs isolated using this method may also relate to the adjusted collagenase concentration. We used a slightly lower concentration (0.1%) than in some conventional protocols (0.15%) [25, 26], which may contribute to better preservation of ECM integrity and reduce enzymatic damage to cell–cell or cell–matrix interactions. This observation is consistent with the previous reports, suggesting that overly harsh enzymatic digestion disrupts MVF structure and reduces their therapeutic potential [6, 32]. Therefore, both digestion kinetics (time and number of cycles) and enzymatic conditions (concentration and activity) are critical parameters for MVF quality.

Compared to previous studies that focused on mechanical fractionation or single-parameter optimization, our study offers a biologically validated, systematically optimized, and clinically adaptable protocol tailored for human lipoaspirate. Moreover, by reducing residual adipose waste and maximizing vascular unit recovery, this method is especially useful when handling limited or valuable clinical samples.

Despite its advantages, our study has several limitations. First, the adipose tissue samples were obtained from relatively healthy, non-obese adult donors; the performance of the method in aged, obese, or metabolically diseased individuals remains to be tested. Second, although in vivo angiogenesis was confirmed in subcutaneous implants, further studies in ischemic injury or wound healing models are needed to evaluate therapeutic efficacy in clinically relevant contexts. Additionally, while enzyme batch consistency and xeno-free compatibility were not addressed in this study, these parameters are critical for downstream clinical translation and regulatory compliance.

Looking forward, this optimized MVF isolation method provides a robust foundation for future development of vascularized tissue constructs. Integration with biomaterials, 3D bioprinting platforms, or injectable hydrogels may further expand its applications in regenerative medicine. Importantly, future work should focus on adapting the protocol for good manufacturing practice (GMP) workflows, validating its efficacy across a broader donor pool, and assessing its impact in disease models. Ultimately, standardized and scalable MVF preparation methods such as the one presented here may facilitate the clinical translation of MVF-based therapies for vascular regeneration, tissue repair, and reconstructive surgery.

## Conclusions

We developed a sequential enzymatic digestion protocol optimized for the efficient isolation of MVFs from human adipose tissue. All procedures were conducted with written informed consent from each participant. This method significantly improves MVF yield, preserves structural integrity and cellular viability, and enhances angiogenic capacity both in vitro and in vivo. The approach addresses key limitations of conventional one-step digestion methods and provides a practical, scalable solution for generating clinically relevant vascularization units. This optimized protocol may facilitate the development of prevascularized tissue constructs for regenerative applications.
